# Serum IL-1ra Is Associated with but Has No Genetic Link to Type 1 Diabetes

**DOI:** 10.3390/endocrines3030048

**Published:** 2022-09-13

**Authors:** Paul M. H. Tran, Fran Dong, Khaled Bin Satter, Katherine P. Richardson, Roshni Patel, Lynn K. H. Tran, Diane Hopkins, Ravindra Kolhe, Kathleen Waugh, Marian Rewers, Sharad Purohit

**Affiliations:** 1 Center for Biotechnology and Genomic Medicine, Medical College of Georgia, Augusta University, 1120 15th St., Augusta, GA 30912, USA; 2 Barbara Davis Center for Childhood Diabetes, University of Colorado Denver, Mail Stop A-140, 1775 Aurora Court, Aurora, CO 80045, USA; 3 Department of Pathology, Augusta University, 1120 15th St., Augusta, GA 30912, USA; 4 College of Allied Health, Augusta University, 1120 15th St., Augusta, GA 30912, USA; 5 Department of Obstetrics and Gynecology, Medical College of Georgia, Augusta University, 1120 15th St., Augusta, GA 30912, USA

**Keywords:** type-1 diabetes, inflammation, cytokines, IL-1 antagonism, IL-1ra, biomarkers

## Abstract

Interleukin-1 antagonism is a proposed biomarker and potential therapy for the delay and/or treatment of type 1 diabetes (T1D). We evaluated the role of circulating interleukin-1 receptor antagonist (IL-1ra) in a prospectively monitored cohort of T1D patients. In order to determine a mechanistic association between IL-1ra and T1D, we performed co-localization analyses between serum IL-1ra protein quantitative trait loci and T1D genome-wide analysis studies. Adjusting for human leukocyte antigen (HLA) genotypes, first degree relative status, gender, and age, serum levels of IL-1ra were lower in subjects who progressed to T1D compared to the controls (*p* = 0.023). Our results suggest that females have higher levels of IL-1ra compared to males (*p* = 0.005). The 2q14.1 region associated with serum IL-1ra levels is not associated with a risk of developing T1D. Our data suggest that IL-1 antagonism by IL-1ra is not an effective therapy in T1D, but IL-1ra may be a biomarker for progression to T1D.

## 1. Introduction

Type 1 diabetes (T1D) is an organ specific autoimmune disease characterized by the destruction of insulin producing pancreatic β-cells, which leads to insulin deficiency and hyperglycemia over time [1]. The destruction of the β-cells is mediated by both innate and adaptive immune cells [2,3]. Along with immune cells, cytokines and chemokines are also involved in the development of T1D autoimmunity via complex interactions between immune cells and β-cells [4,5]. Notably, chemokines [6] and cytokines such as interleukin (IL)-1β [7], IL-1 receptor antagonist (IL-1ra) [8], and monocyte chemoattractant protein (MCP)-1 have shown involvement in β-cell death.

The Hvidøre Study group found that higher IL-1ra levels are associated with preserved beta-cell function [9,10]. In a small sample size (n = 103), Schloot et al. (2007) showed that serum levels of IL-1ra decreased in T1D patients [8]. In a larger case–control study (n = 4424), Purohit et al. (2015) reported that serum Il-1ra [11] decreased in T1D subjects compared to controls [8,10–12]. Currently, there are no longitudinal studies that have evaluated IL-1ra serum levels in individuals from birth to the development of clinical diabetes.

IL-1ra inhibits the activity of IL-1 by competing for the IL-1 receptor and regulates inflammation. The Il-1 signaling pathway is typically pro-inflammatory through the signaling receptor IL-1 receptor type 1 (IL-1R1) and its ligands, IL-1β and IL-1α. IL-1ra is a competitive inhibitor of Il-1 signaling. The global knockout of the IL-1R in non-obese diabetic (NOD) mice led to a significant delay in diabetes development [13]. While the administration of Il-1ra can inhibit disease recurrence after syngeneic pancreatic islet transplantation in NOD mice [14], it is not able to decrease diabetes incidence as a single agent in these mice [15]. Trials in humans showed similar findings where both IL-1 blockade and recombinant IL-1ra (anakinra) were not effective single agents for recent-onset T1D [16]. Interestingly, the administration of anakinra did reduce serum interleukin 8 and monocyte-specific CD11b integrin expression, suggesting a reduction in mononuclear cell trafficking to inflammatory sites [17]. NOD mouse studies have suggested a role for IL-1 blockade in synergizing with anti-CD3 for T1D prevention [15].

We validate the association between a decrease in serum IL-1ra progression to T1D in a longitudinal cohort and use human and mouse genetics to explore a potential causal role for IL-1ra in the progression to T1D.

## 2. Materials and Methods

### 2.1. Human Subjects

The serum samples (n = 312) analyzed were part of the Diabetes Autoimmunity Study in the Young (DAISY, Denver, CO, USA) study, conducted between January 1994 and November 2006, which followed 2547 children at increased risk for T1D [18–20]. DAISY study defined increased risks for T1D as either the possession of a high diabetes risk HLA genotype or having a sibling or parent with T1D [18–20]. Islet autoantibodies (IA) to insulin, GAD, IA-2, and ZnT8 were measured by a radio-binding assay. Persistence IA was defined as testing positive for one or more islet autoantibodies on two or more consecutive visits or being diagnosed with T1D within a year using the American Diabetes Association (ADA) criteria [21]. The sex and age of the first serum sample, first-degree relative status (FDR) of T1D, as well as HLA-DQB1 genotypes for all subjects are summarized in Table 1. The study was conducted according to the Declaration of Helsinki (1997), and it was approved by the institutional review board at Augusta University (#611249). All subjects or their parents provided signed consent before participating in the study.

Blood samples were collected in serum separator tubes (BD Biosciences) and allowed to clot for 30 minutes at room temperature. Aliquots of plasma and serum were prepared immediately after phlebotomy into wells of 96-well plates (150 μL/well) to create master plates. Daughter plates were then created by pipetting 5–25 μL of serum/well to avoid repeated freeze/thaw for all samples.

### 2.2. Fluorescent Immuno-Assay Measurement of IL-1ra

Multiplex fluorescent immunoassay (Millipore, Billerica MA, USA) was used to measure serum levels of IL-1ra as per conditions recommended by the manufacturer. Pre-diluted (5×) serum samples were mixed with capture antibodies immobilized on polystyrene beads for one hour. The unbound serum was removed from beads, which were then washed three times and further incubated with a biotinylated detection antibody cocktail for another hour. The detection antibody was removed by washing beads twice. The beads were then incubated with phycoerythrin-labeled streptavidin for another thirty minutes. After a final wash, the beads were washed and suspended in 60 μL of wash buffer. The median fluorescence intensity (MFI) was captured on a FlexMAP 3D array reader (Millipore, Billerica, MA, USA) with the following settings: events/bead: 50; minimum events: 0; flow rate: 60 μL/min; sample size: 50 μL and discriminator gate: 8000–13,500.

Before profiling, serum dilutions were optimized by performing assays at different serum dilutions to ensure that the majority of the data falls within the linear range of the standard curve [11].

### 2.3. Data Acquisition

DAISY phenotype data were provided by MR. IL-1ra pQTL data were downloaded from Zenodo (https://zenodo.org/record/2615265#.YGs9GEhKjYk) accessed on 12 January 2021 [22]. IL-1ra GWAS summary statistics was downloaded from NHGRI-EBI GWAS catalogue from http://ftp.ebi.ac.uk/pub/databases/gwas/summary_statistics/GCST90014001-GCST90015000/GCST90014023 accesssed on 12 January 2021 [23].

### 2.4. Statistical Analysis

All statistical analyses were performed using the R language and environment for statistical computing (R version 3.16; R Foundation for Statistical Computing; www.r-project.org) and SAS version 9.4 (SAS Institute, Cary, NC, USA). All *p*-values were two-tailed, and a *p* < 0.05 after adjusting for multiple testing was considered statistically significant.

Protein concentrations were estimated using a regression fit to the standard curve with known concentrations included on each plate using a serial dilution series [11]. The data were subjected to several quality control steps before further analysis: (1) Individual wells with low bead counts (below 30) or high bead CV (above 200) were flagged for exclusion. (2) Replicate wells with coefficient of variation > 25% were not included in further analyses. Two pooled QC samples, representing controls and T1D patients, were included on each plate in triplicate. Data from these wells were used for plate-to-plate normalization. The limits of detection were determined using the MDD data provided by the manufacturer (IL-1ra: 2.9 pg/mL), and limits of quantitation were determined using the lower saturation end of standard curves. Wells below the limit of detection or limit of quantitation were flagged for exclusion. If any of the wells failed QC steps, the subject’s data were excluded from further analysis.

Serum values not in the range of (Q1 −1.5 IQR) and (Q3 +1.5 IQR) are considered outliers and were set as missing for downstream analysis. Categorical variables were analyzed using Pearson χ^2^ tests. Continuous variables were tested using the ANOVA test for differences in means or the Kruskal–Wallis Test for differences in medians. Because IL-1ra data are not normally distributed, a square-root transformation was performed to normalize the data. Linear mixed models (PROC mixed) are used to compare the difference in transformed IL-1ra levels among the three study groups, adjusting for age, gender (female vs. male), and HLA genotype ((DR3/4 or DR4/4 or DR4/X) vs. (DR3/3 or DR3/X or DRX/X) and FDR status (FDR vs. GP) with a random intercept in the model35. We decide to use the model with linear line over age as the final model for apparent interpretation of the data.

Gaulton-GWAS data were converted to GrCh 38 coordinates using liftover, dowloaded from https://genome-store.ucsc.edu/ (accessed on 12 January 2022) with a corresponding chain file downloaded from http://hgdownload.soe.ucsc.edu/downloads.html#source_downloads (accessed on 12 January 2022). LocusZoom was used to generate both Manhattan and LocusZoom plots. All *p*-values were two-tailed, and a *p* < 0.05 after adjusting for multiple testing was considered statistically significant.

## 3. Results

### 3.1. Clinical and Demographic Characteristics of DAISY Cohort

We analyzed 312 subjects, consisting of 222 control subjects, 49 progressor subjects, and 41 non-progressor subjects. Progressors were children who progressed to T1D. Non-progressors were children with at least two consecutive positive visits for islet autoantibodies (insulin, glutamate dehydrogenase-65, ZnT8, and insulinoma antigen-2A) but did not develop T1D. The control subjects are subjects who neither developed islet autoantibodies nor T1D. Serial serum samples (mean 9 (range 3 to 28)) were collected from each subject. The median number of samples per subject in each study group included the following: 7 samples in the control group, 12 samples in the non-progressors, and 13 samples in the progressors. The average follow-up time for the DAISY subjects was 16.91 years with a range of 1.98 to 31.00 years. The mean ± SD age at the appearance of islet autoantibodies was 4.57 ± 3.22 years in progressors and 7.64 ± 4.24 years in non-progressors. The mean age of T1D diagnosis in progressors was 12.32 ± 5.67 years. Ages of the first sample, gender, HLA genotype, and FDR status were similar among the three study groups. Results are summarized in Table 1.

### 3.2. Serum Levels of IL-1ra Are Associated with Progression to T1D

**Figure 1.**
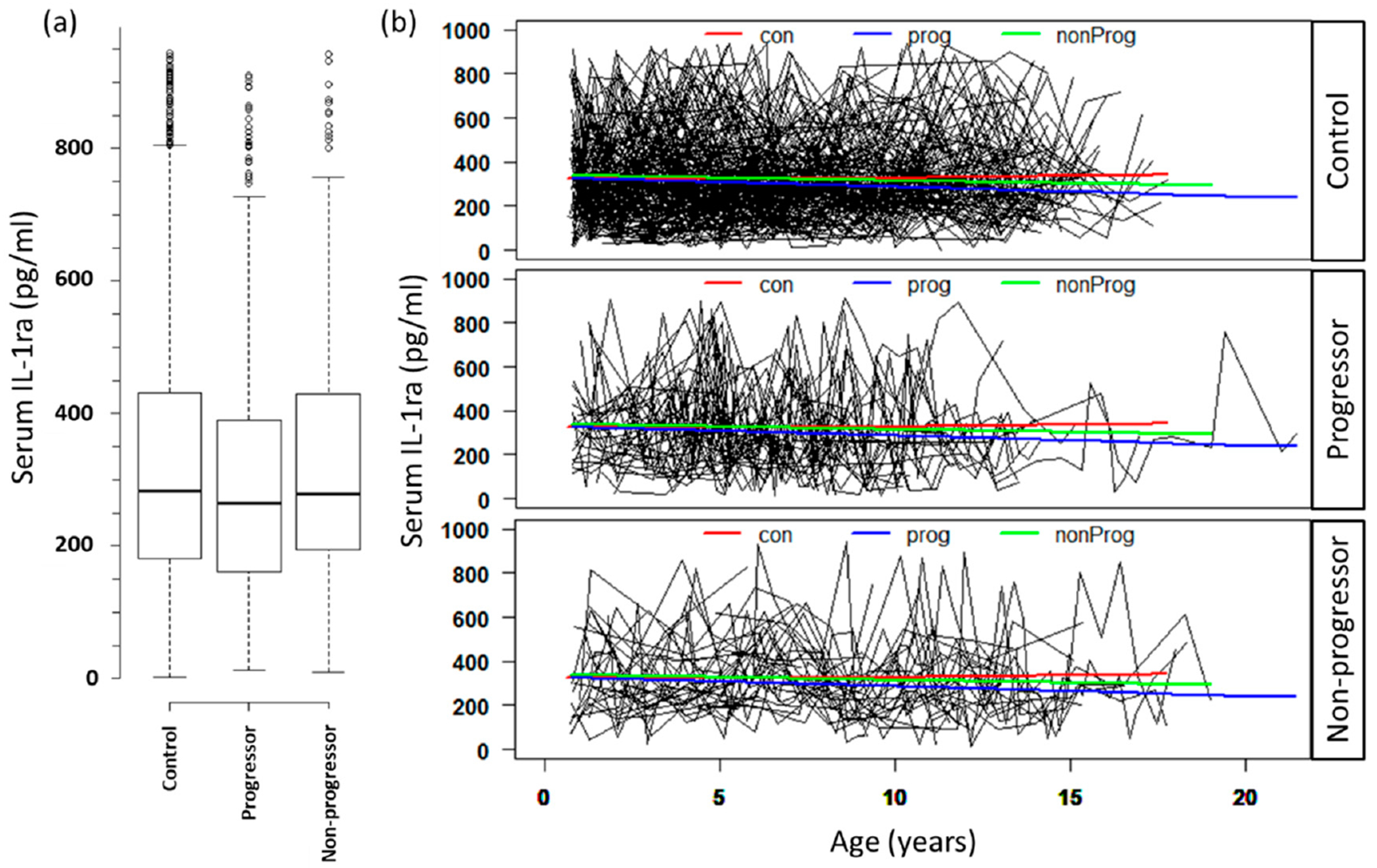
Distribution of IL-1ra levels in three groups. (**a**) Boxplot showing differences in serum level of IL-1ra in three groups in the study. (**b**) Spaghetti plots showing IL-1ra level in serum over age in subjects from three groups. IL-1ra levels were measured in longitudinally collected samples over time. Average number of samples per subjects was 9 with a range of 3 to 28 samples. Serum IL-1ra level (pg/mL, *y*-axis) is plotted against the age of collection of sample (*x*-axis). A smooth spline was plotted for con: controls (red line); prog: progressor (blue line); and nonProg: non-progressor (green line). The progressor group shows a decline in serum IL-1ra from the start and decreased markedly as the age progresses.

Using linear mixed models, we next tested whether the mean IL-1ra level differed among the three study groups and adjusted for HLA genotype, FDR status, gender, and age (Table 2). IL-1ra level did not differ by HLA group and the level of IL-1ra did not change over time (age). A comparative analysis of serum level of IL-1ra over age among three groups (controls (n = 222), progressors (n = 49), and non-progressors (n = 41)) is presented in Figure 1. IL-1ra levels were significantly lower in progressors compared to the controls (*p* = 0.023). Among progressors (n = 49), IL-1ra levels decreased significantly faster in subjects who progressed to T1D before age of 11 years (β= −0.48 ± 0.16, *p* = 0.003) vs. subjects who progressed after 11 years of age (β= −0.08 ± 0.07, *p* = 0.275). Females had higher IL-1ra levels compared to males (*p* = 0.005). Those with first-degree relatives with T1D had higher levels of IL-1ra compared with the general population (*p* = 0.001).

### 3.3. Serum IL-1ra Levels Are Not Genetically Associated with T1D

**Figure 2.**
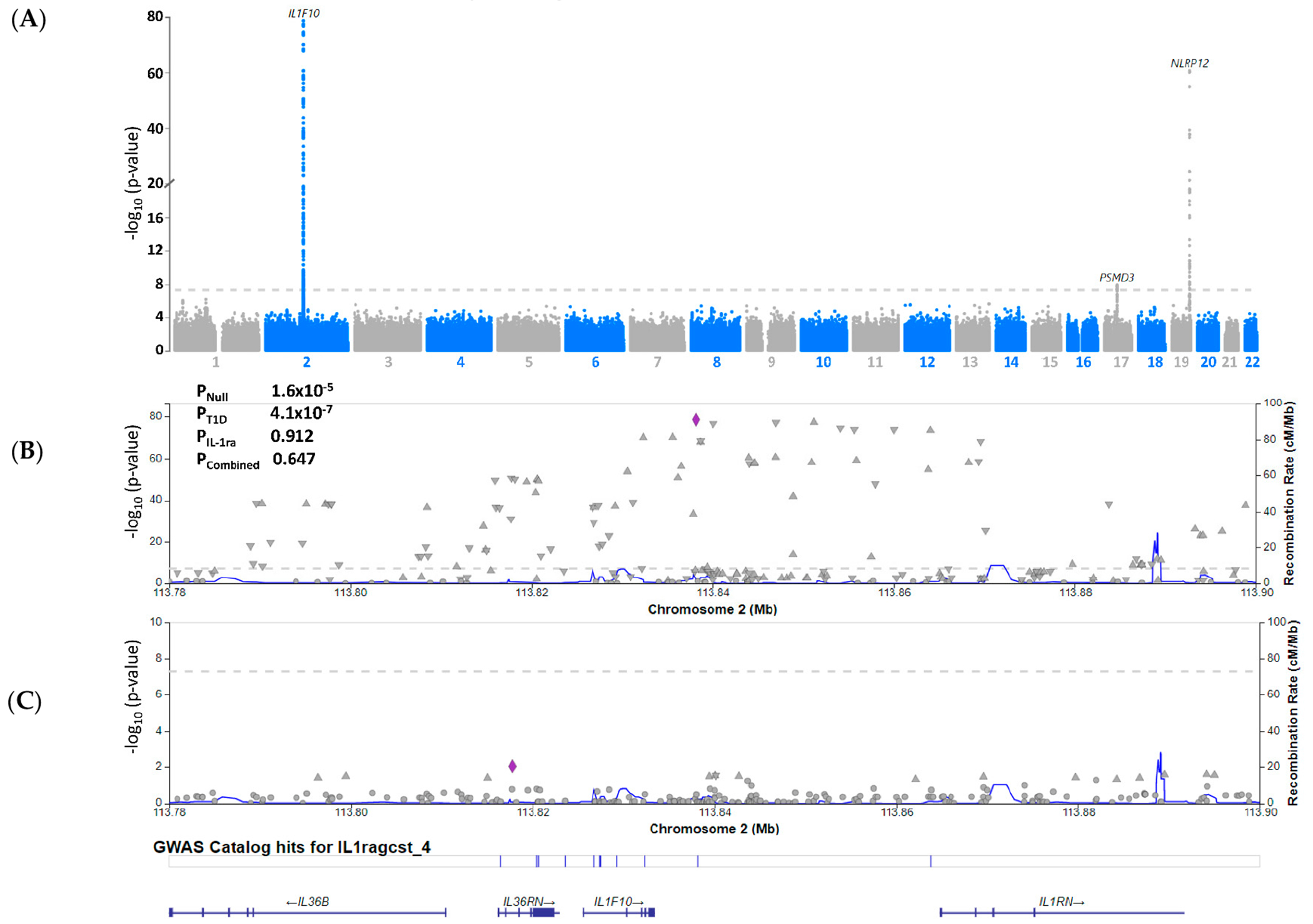
Serum IL-1ra levels are not genetically associated with type 1 diabetes (T1D). (**A**) Manhattan plot of genetic loci associated with serum IL-1ra levels from Folkersen et al. [22] protein quantitative trait loci study. (**B**) LocusZoom plot of SNPs associated with serum IL-1ra levels in the 2q14.1 region from Folkersen et al. [22] protein quantitative loci (pQTL) study. Association between genetic locus and pQTL was performed using Coloc analysis: P_Null_: coloc *p*-value for null hypothesis; P_T1D_: coloc *p*-value for genetic locus associated with T1D; P_IL-1ra_: coloc *p*-value for association of IL-1ra protein levels; and P_combined_: coloc *p*-value for the combined analysis of genetic risk and IL-1ra pQTL. (**C**) LocusZoom plot of SNPs associated with T1D in the 2q14.1 region from Chiou et al.’s study.

We assessed the association between serum IL-1ra levels and T1D for a genetic basis by performing colocalisation analyses on the summary statistics data for IL-1ra pQTL [22] and T1D GWAS [23]. IL-1ra pQTL data showed that genetic loci 2q14.1 (IL1F10) and 19q13.42 (NLRP12) are associated with serum IL-1ralevels (Figure 2A). When we compared this with T1D GWAS summary statistics [23], we found no colocalized loci between the two analyses (Figure 2B).

## 4. Discussion

We show that after adjusting for co-variates such as sex, age, first-degree relatives and high-risk HLA genotypes, serum levels of IL-1ra decreased in progressors to T1D compared to the controls. In addition, we found that serum IL-1ra levels were elevated in females compared to males and first-degree relatives compared to the general population.

The decreased levels of IL-1ra observed in the prospectively monitored DAISY cohort validates our previous report showing serum IL-1ra levels are reduced in T1D subjects [11]. Reduced levels of IL-1ra have been observed in T1D patients in earlier studies [8] and type 2 diabetes [24], suggesting that decreased levels of IL-1ra may be a predictive biomarker for T1D.

We observed that females have higher serum levels of IL-1ra compared to males. Previous reports have shown that females produce increased levels of inflammatory cytokines [25]. Additionally, gonadal hormones have been implicated in the higher serum level of several cytokines, including IL-1ra [26,27]. There are higher incidence rates of autoimmune diseases in females compared to males [21]. However, the male-to-female relative incidence of T1D (1.7:1) is the opposite of other autoimmune diseases [28].Our finding of higher serum IL-1ra levels in females suggest that differences in IL-1ra levels are driven by sex hormones, which may play a role in the sexual dimorphism of autoimmune diseases [29]. In the pQTL analysis included in this study, only autosomes were studied to determine the effect of protein expression on GWAS, so genetic associations with the sex chromosome could not be assessed. Additionally, T1D GWAS have not been conducted to identify sexually dimorphic loci, which may uncover loci co-localized between serum IL-1ra levels and T1D. Despite this, our mixed model analysis found that the IL-1ra serum levels are associated with progression to T1D, even accounting for sex, suggesting a role distinct relative to the sexual dimorphism of IL-1ra levels.

We observed higher levels of IL-1Ra in those with FDR with T1D compared to GP. Several explanations could exist for this. (1) Shared environmental factors among first degree relatives who develop T1D are also associated with serum IL-1Ra. (2) There is a genetic correlation between T1D and IL-1Ra, which is not appreciated on the current genotyping platforms used in this study, possibly from rare SNPs or in the sex chromosomes. (3) There is a combined genetic–environment interaction effect that is associated with both T1D and serum IL-1Ra levels. Our findings suggest a correlation between genetic and environmental factors that overlap between FDRs of T1Ds and higher levels of IL-1Ra.

Our data suggest that IL-1Ra level’s decreases much faster among early (<11 years old) progressors compared to late progressors (>11 years old), which is interesting since young progressors have more rapid beta-cell loss compared to older progressors. IL-1Ra levels may be a surrogate marker for the loss of beta-cell function, although more studies in larger cohorts are required to assess the clinical utility of this finding.

## 5. Conclusions

Our results suggest that while serum IL-1ra levels are associated with progression to T1D, IL-1ra does not play a causal role in this progression based on human genetic co-localization analysis. This is in agreement with pre-clinical and clinical trials that have shown minimal effects of recombinant interleukin-1 receptor antagonist (anakinra) as a single agent for T1D prevention [30–32].

## Figures and Tables

**Table 1.** Baseline characteristics of the DAISY study participants (n = 312).

Subject Characteristics		Control (n = 222)	Non-Progressor (n = 41)	Progressor (n = 49)	*p* Value
Age of first sample, median (IQR)		1.26 (0.78–2.58)	1.29 (0.85–3.09)	1.28 (0.80–2.73)	0.7
HLA genotype, n (%)	DR3/3 or DR3/X or DRX/X	90 (40.54)	13 (31.71)	12 (24.49)	0.08
	DR3/4 or DR4/4 or DR4/X	132 (59.46)	28 (68.29)	37 (75.51)	
FDR status, n (%)	GP	97 (43.69)	16 (39.02)	17 (34.69)	0.48
	FDR	125 (56.31)	25 (60.98)	32 (65.31)	
Gender, n (%)	Female	97 (43.69)	22 (53.66)	24 (48.98)	0.45
	Male	125 (56.31)	19 (46.34)	25 (51.02)	

**Table 2.** Multivariate mixed model regression analysis of IL-1ra with age, gender, HLA type, first degree relative, and status.

Effect		Estimate	Standard Error	*p*-Value
Intercept		16.18	0.35	<0.0001
Age		0.01	0.03	0.64
Gender	Female vs. Male	0.72	0.26	0.005
HLA	DR3/4 or DR4/4 or DR4/X vs. DR3/3 or DR3/X or DRX/X	0.09	0.28	0.75
First-degree relative	YES vs. NO	0.88	0.27	0.001
Progression to T1D	Non-progressor vs. Control	−0.2	0.36	0.58
	Progressor vs. Control	−0.74	0.33	0.023
	Progressor vs. Non-progressor	−0.54	0.43	0.2

## Data Availability

The data that support the findings of this study are available from the corresponding author, SP, upon reasonable request.
